# Examining and Interpreting Doi Plot Asymmetry in Meta‐Analyses of Randomized Controlled Trials

**DOI:** 10.1111/jebm.70063

**Published:** 2025-08-29

**Authors:** Luis Furuya‐Kanamori, Xanthoula Rousou, Polychronis Kostoulas, Suhail A. R. Doi

**Affiliations:** ^1^ UQ Centre for Clinical Research Faculty of Health, Medicine and Behavioural Sciences The University of Queensland Herston Australia; ^2^ Laboratory of Epidemiology, Applied Artificial Intelligence & Biostatistics School of Health Sciences Faculty of Public and One Health University of Thessaly Karditsa Greece; ^3^ Department of Population Medicine College of Medicine QU Health Qatar University Doha Qatar

**Keywords:** Doi plot, Eggers *p*, funnel plot, LFK index, meta‐analysis, publication bias

## Abstract

Systematic reviews and meta‐analyses are considered the highest level of evidence, but their reliability can be undermined by publication bias. Traditional methods for assessing publication bias, such as funnel plots and *p*‐value‐based tests (e.g., Egger test), have notable limitations, including reliance on subjective interpretation and dependence on the number of studies included in a meta‐analysis (*k*). The Doi plot and LFK index offer promising alternatives, providing improved visualization and quantification of plot asymmetry. This study revisits the application of the Doi plot and LFK index for detecting publication bias, addresses recent criticisms, and evaluates their performance compared to *p*‐value‐based methods using simulation study. Simulations included scenarios with varying study numbers (*k* = 5, 10, 20, 50), study sample sizes (small, large), and simulated bias level (*ρ* = 0, –0.3, –0.5, –0.9) generated using the Copas selection model. Diagnostic performance metrics (i.e., sensitivity and specificity) were estimated and compared for the LFK index and Egger test. The LFK index exhibited consistent higher sensitivity across varying *k* and simulated bias levels. In contrast, the Egger test was highly dependent on *k*, with sensitivity declining sharply in small meta‐analyses (*k* < 20). Specificity of the LFK index adjusted with random error, while Egger test specificity remained fixed at ∼90%. The Doi plot and LFK index effectively address the limitations of traditional methods, offering robust *k*‐independent performance and more reliable detection of publication bias. These findings support a transition to the Doi plot and LFK index for publication bias assessment in meta‐analyses.

## 1 Background and Approaches to Assess Publication Bias

Systematic reviews and meta‐analyses provide the highest level of evidence by synthesizing all the evidence on a specific topic, thereby providing reliable conclusions that guide clinical and policy decisions [1]. However, the robustness of conclusions drawn from meta‐analyses rely heavily on the completeness of the evidence included. When certain studies are excluded, whether intentionally or inadvertently, the resulting evidence can become biased and potentially misleading. Among the various factors that may limit the inclusion of studies in meta‐analyses, publication bias remains a significant concern. Publication bias occurs when the likelihood of a study being published is influenced by the magnitude or direction of its findings, leading to an underrepresentation of studies with less significant or unfavorable results [2]. The selective inclusion of studies with positive findings not only affect the perceived efficacy/effectiveness of interventions, but also have broad implications for evidence‐based practice, as they may skew the understanding of both the risks and benefits associated with a particular intervention or exposure.

Several approaches, both graphical and statistical, have been developed to detect and quantify publication bias [3]. Among the most widely used methods are graphical techniques such as funnel plots, which visually assess the (a)symmetry of study effect sizes against a form of precision. The funnel plot has faced significant criticism due to its reliance on subjective visual interpretation [4], often leading to misleading conclusions about the presence or absence of asymmetry [5]. Furthermore, its utility is limited in specific contexts, such as with prevalence data [6, 7] and when standardized mean differences are plotted against standard errors [8]. Additionally, changes in the definition of precision and/or effect measures have been found to alter the conclusions about funnel plot asymmetry in up to 86% of meta‐analyses [9].

These limitations underscore the need for alternative methods [10, 11], and to address the inherent shortcomings of the funnel plot, the Doi plot was developed [12]. This innovative approach modifies the normal quantile plot, plotting absolute *Z*‐scores in reverse order on the *Y*‐axis and effect sizes on the *X*‐axis. The smallest absolute *Z*‐score serves as the reference point, creating a tip, with a perpendicular line dividing the plot into two regions. While the Doi plot is a scatterplot of precision versus effect size, the studies form the limbs of the plot rather than the body, providing a distinct visual structure. This design differs significantly from the funnel plot, offering a clearer and more intuitive framework for visual interpretation.

Given the problems with visual interpretation of the funnel plot, quantification became a means to better “detect” asymmetry. One measure could have been the intercept of the regression [13] but is rarely used because it lacks a clear interpretation. It estimates the average of study‐specific standardized deviates, which does not account for the shape of the deviates [3]. Meta‐analysts therefore usually do not report the magnitude of the intercept, but just the *p*‐value of Egger's regression test [13] or similar tests such as Begg [14], Peters [15], and Thompson‐Sharp [16] tests (hereafter referred to as *p‐*value‐based methods). These methods rely on statistical significance testing to evaluate the association between an effect estimate and its precision. Typically, a *p*‐value below a set threshold (usually <0.1) suggests the presence of asymmetry. However, as with other significance tests, these *p*‐values are dependent on the number of studies included in a meta‐analysis, meaning that a symmetrical set of studies could yield a low *p‐*value if the number of studies is large. This fundamental limitation was demonstrated in empirical analyses and a controlled experiment where the same level of asymmetry was maintained while the number of studies varied [17] resulting in changes in the *p*‐value.

While the Doi plot offers improved visual clarity, quantifying asymmetry remains crucial for several reasons. It allows for comparisons between meta‐analyses [3], and quantifies the degree of asymmetry required to provide robust evidence of bias [5]. Therefore, the LFK index [12] was developed to quantify the (a)symmetry of the Doi plot. The LFK index measures the difference in area under the Doi plot between the two regions on either side of the most precise study. In a symmetric plot, these areas would be roughly equivalent, resulting in an LFK index close to zero. Unlike *p*‐value‐based methods, the LFK index is an *effect size measure* (analogous to a mean difference) rather than a statistical test, making it independent of the number of studies when assessing asymmetry. This independence from the meta‐analytic number of studies of the LFK index has been confirmed in the experiment explained above [17].

While the Doi plot and LFK index have been widely adopted, with over 900 citations, it has not been immune to criticism [18]. In this paper, we revisit the application of these novel methods for assessing publication bias, while addressing key conceptual misunderstandings and recent criticisms. Furthermore, we conduct simulation studies to demonstrate how the LFK index compares to *p*‐value‐based tests in the detection of publication bias.

## 2 Conceptual Issues Raised by Schwarzer et al

Two misconceptions regarding the LFK index have recently come to light in the literature [18].

First, it has been referred to as the “*LFK index test*” as if it is a *p*‐value‐based test. The authors that suggested this also backtrack when they say that “…*we do not understand the distinction between the LFK index and ‘p value‐driven’ methods (aka statistical tests).”* This clearly is a key misconception, as outlined in the seminal paper for these methods [12] and described earlier. The LFK index is an *effect size* because it is the magnitude of the difference between two regions on the Doi plot. This makes the LFK index similar to any other magnitude of effect in common use such as risk difference or mean difference. Working on their “assumption” of the statistical test status for the LFK index, these authors further state that *“the major difference between the LFK index and established statistical tests is that the critical value for the LFK index lacks any justification and that the false positive rate is unpredictable.”* There is no critical value for an effect size, except its mathematical limits, and just as the mean difference is bounded between ±infinity (with 0 as its null value), so too the LFK index can run through ±infinity (at least theoretically) with zero as its null value. Because they misrepresent the LFK index as a statistical test, the authors go on to state that “*a reliable assessment of the extent of asymmetry based on the value of the LFK index is fruitless as long as the approximate distribution of the LFK index under the assumption of symmetry is unknown*.” However, we respond to this claim much like Vineet Tiruvadi, who aptly stated: “*If you start with the wrong framework then the ability to do complex analyses may seem like it's giving insight, but what you're mostly doing is studying how wrong your framework is”* [19].

The second misconception raised was that evaluation of quantitative assessments of study asymmetry in a diagnostic sense is wrong because it “*implies that the cut‐off to detect asymmetry is varied*.” These assessments are on a continuous scale, and publication bias is a binary outcome. If we view the quantitative assessments as a “test” of the outcome, we can certainly use diagnostic terminology. By dichotomizing the assessments at predefined thresholds (say ±1 for LFK and 0.1 for *p*‐value‐based tests), we can derive all the necessary diagnostic indices. There is a parallel between diagnosis and errors in statistical testing, thus we will continue to use diagnostic terminology with a binary test (cut‐offs –1 for LFK and 0.1 for *p*‐value‐based tests) when we present comparisons under simulation.

## 3 Reevaluation of an Independently Conducted Simulation Study

To replicate the simulation study by Schwarzer et al. [18], modifications to the simulation parameters were made to better reflect the typical meta‐analyses encountered in real‐world, as summarized in Table 1. The modified simulation code in R (based on Schwarzer et al.) is provided in the Supplementary Material (S1).

**TABLE 1:** Parameters used in the simulation studies.

	Schwarzer et al.	Replication of Schwarzer et al.
Number of meta‐analyses per simulation scenario	10,000	1000
Number of studies (*k*)	10, 20, 50, 100	5, 10, 20, 50
Mean (*ue*), standard deviation (*sde*) in the experimental group	*ue* 0, 1, 2; *sde* 1, 1.5, 2	*ue* 0; *sde* 1
Mean (*uc*), standard deviation (*sdc*) in the control group	*uc* 2; *sdc* 1	*uc* 2; *sdc* 1
Square root of the between‐study variance (*τ*)	0, 0.25, 0.5	0
Distribution of sample size of studies	*Small*: Log‐normal *Large*: Reported as uniform but **not** uniform (Figure 1)	*Small*: Log‐normal *Large*: Log‐normal
Copas bias parameter (*ρ*)	0, –0.5, –0.9	0, –0.3, –0.5, –0.9
Asymmetry by Egger (*p*‐value)	< 0.05	< 0.1
Asymmetry by LFK index	< –1	< –1

It is important to note that graphical/quantitative techniques described herein do not assess publication bias per se. As pointed out by Sterne et al. [20]. there are other potential sources of asymmetry, which can be summarized into systematic and random error (Box 1). Therefore, our primary aim in replicating this simulation was to demonstrate why the performance of the LFK index vis‐à‐vis the Egger test was reported differently by Schwarzer et al. and what the correct interpretation should be.

**Box 1. Main sources of potential asymmetry of graphical techniques**

1. Reporting biases (including publication bias)—Systematic error
2. True heterogeneity—Systematic error
3. Small study effects—Systematic error
4. Artifacts of the data and chance variation—Random error

To isolate the effects of publication bias, the most notable change in our simulations was the exclusion of between‐study heterogeneity (*τ^2^*), a well‐known contributor to plot asymmetry (Table 1) [20, 21]. This approach ensures clearer evaluation of the methods under controlled conditions and focuses specifically on the ability of the LFK index and Egger test to distinguish asymmetry due to publication bias from that due to random error.

The number of studies (*k*) in the simulation was adjusted from the original values of 10, 20, 50, and 100 to a more realistic range of 5, 10, 20, and 50. This adjustment was made to remain realistic as most meta‐analyses are small given the reported distribution of studies included in 47,481 meta‐analyses (with at least 3 studies) from 4359 Cochrane Reviews, which reported a median of 5 studies (interquartile range: 3–12).

**FIGURE 1:**
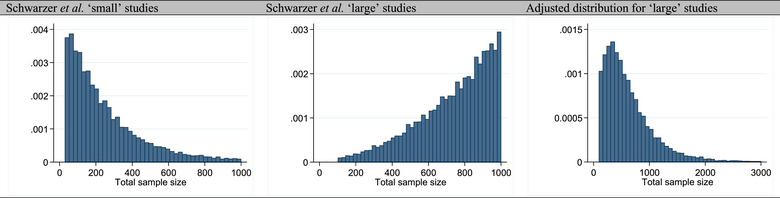
Distribution of total sample size for scenarios with 20 studies per meta‐analysis and no simulated publication bias (Copas bias parameter [*ρ*] = 0).

Additionally, we noted that the distribution of the total sample sizes for “small” studies followed a log‐normal distribution, while the distribution for “large” studies was reported as “uniform,” but the simulated distribution was not uniform and found to be negatively skewed. This faulty simulation had the undesirable effect that study sizes within simulated meta‐analyses were similar and thus the Copas bias parameters had minimal introduction of asymmetry. To avoid this pitfall and to ensure consistency and better reflect real‐world meta‐analytic scenarios, where smaller studies are more prevalent and larger studies are relatively rare, the distribution of “large” studies was aligned to that of “small” studies to also follow a log‐normal distribution (Figure 1).

Other changes were made primarily to simplify and expedite the simulation process. The number of meta‐analyses per simulation scenario was reduced from 10,000 to 1000, as convergence was observed with fewer iterations and to balance computational efficiency with practical utility. Additionally, the number of scenarios based on variations in the mean (*ue*) and standard deviation (*sde*) in the experimental group was reduced from 9 to only 1. Furthermore, the *p*‐value‐based tests were restricted to the Egger test, as it is the most commonly utilized method for assessing funnel plot asymmetry.

The Copas bias parameters (*ρ* = 0, –0.5 [moderate], –0.9 [severe]) were retained, but an additional value of –0.3 [mild] was introduced to provide a milder level of bias. *ρ* = 0 corresponded to no bias. Since only negative bias was simulated, LFK index values below –1 were considered indicative of asymmetry. For the Egger test, a *p*‐value threshold of 0.1 (which is the norm), was applied instead of 0.05.

### 3.1 Evaluation of the Performance of the *p*‐Value‐Based Test and LFK Index

The gold standard for the diagnostic indices was the simulated bias scenario: those meta‐analyses within *ρ* = 0 scenarios were tagged as the gold standard negative (no bias), and those meta‐analyses within *ρ* ≠ 0 (*ρ* of –0.3, –0.5, or –0.9) scenarios were tagged gold standard positive (bias present).

The performance measures included:

1. Sensitivity (also referred to as true‐positive rate or power by Schwarzer et al.) indicates the proportion of meta‐analyses with the LFK index < –1 or Egger test *p‐*value <0.1 for meta‐analyses where the gold standard was positive.
2. Specificity (equivalent to 1 – false‐positive rate or 1 – type I error as described by Schwarzer et al.) indicates the proportion of meta‐analyses in scenarios without simulated publication bias (*ρ* = 0) where LFK index ≥ –1 or Egger test *p‐*value *≥* 0.1 (i.e. where the gold standard was negative).

Each diagnostic assessment compared 1000 meta‐analyses with *ρ* = 0 and 1000 meta‐analyses with *ρ* ≠ 0. The evaluations were conducted across four levels of *k*, three levels of *ρ*, and two sample sizes (small and large) for a total of 24 diagnostic assessments of each method.

### 3.2 Evaluation of the Effect of *k*

Prediction of the binary quantitative method beyond its threshold (LFK < –1 or *p*‐value < 0.1) from two predictors—that is, Copas bias parameter (*ρ*) and *k* was modeled using logistic regression without product terms. Margins plots were examined after each model to demonstrate the impact of *ρ* and *k* on the predicted probability of the quantitative assessment suggesting asymmetry.

## 4 Simulation Findings

### 4.1 Sensitivity

For meta‐analyses of “small” studies and the scenario with mild bias (*ρ* = –0.3), both the LFK index and Egger test had similar sensitivity (∼80%) at *k* = 50. However, as *k* decreased, the sensitivity declined for both, with a much steeper decline observed for the Egger test, which dropped to 15% at *k* = 5, while the LFK index sensitivity dropped only slightly to ∼60%. In scenarios with moderate bias (*ρ* = –0.5), sensitivity for both methods were ∼100% at *k* = 50, and declined to 26% for Egger test, whereas the LFK index dropped only slightly to about 72% for *k* = 5 (Table 2).

**TABLE 2:** Performance of the LFK index and Egger test in detecting publication bias.

			*ρ* = 0		*ρ* = –0.3		*ρ* = –0.5		*ρ* = –0.9	
		Number of studies per meta‐analysis	LFK index FP%	Egger test FP%	LFK index TP%	Egger test TP%	LFK index TP%	Egger test TP%	LFK index TP%	Egger test TP%
***Small studies***	**TP (%) for *ρ* ≠ 0 &** **FP(%) for *ρ* = 0**	***k* = 5**	**34.3**	9.2	**57.7**	15.1	**72.2**	26.5	**93.4**	72.4
		***k* = 10**	**28.5**	9.5	**64.0**	24.3	**82.6**	53.6	**99.3**	97.7
		***k* = 20**	**16.3**	9.2	**70.5**	44.1	**92.5**	80.9	**100.0**	100.0
		***k* = 50**	**6.1**	9.4	**79.6**	78.6	**99.0**	99.2	**100.0**	100.0
***Large studies***	**TP (%) for *ρ* ≠ 0 &** **FP(%) for *ρ* = 0**	***k* = 5**	**29.2**	9.2	**53.3**	16.5	**66.8**	27.5	**88.8**	75.2
		***k* = 10**	**24.1**	11.1	**61.1**	27.7	**83.9**	58.0	**98.5**	98.4
		***k* = 20**	**14.8**	9.4	**67.3**	46.8	**92.0**	85.9	**99.8**	100.0
		***k* = 50**	**4.6**	11.5	**73.3**	82.2	**99.0**	99.4	**100.0**	100.0

*k* number of studies in the meta‐analyses; *ρ* Copas bias parameter quantifying the degree of simulated publication bias (more negative values indicate stronger bias). Figure 3 depicts the same data in this table, but the margins plot depicts the modelled proportions from logistic regression that apply to the whole dataset which are much closer together while this table has the raw proportions. The differences in TP or FP by *k* for LFK (in bold) between the two approaches are possibly due to artifacts of the sample stratification process (random error) and therefore are not seen in the modelled results depicted in Figure 3 for LFK but not so for Egger's *p*. If the goal is to obtain a model based estimate that applies across the whole dataset, logistic regression (Figure 3) is preferable. Note specificity = 100 – FP% and sensitivity = TP%.

**FIGURE 2:**
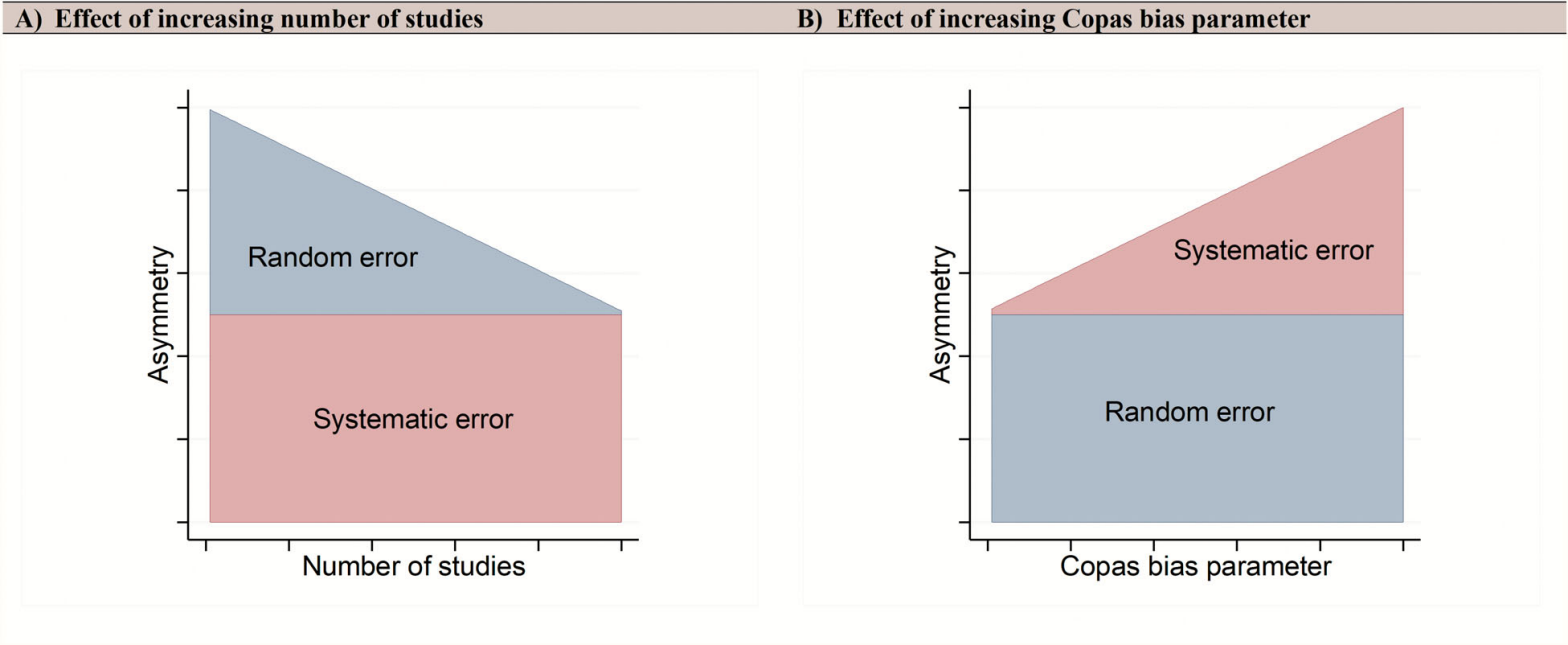
Schematic (not to any scale) depicting the expected effect of (A) number of studies in a meta‐analysis (*x*‐axis) and (B) Copas bias parameter value (*x*‐axis) on the contribution of random (blue) and systematic (red) error to the degree of asymmetry (*y*‐axis).

The decrease in LFK sensitivity is due to increasing random error related asymmetry as *k* decreases (Box 1 and Figure 2). In contrast, the much steeper decline in Egger test sensitivity is attributable to the additional influence of *k* on the threshold of *p*‐value‐based methods (see “Effect of *k*” below) making it a much less reliable measure.

### 4.2 Specificity

For meta‐analyses of “small” studies both LFK index and Egger test had similar specificity (∼90%) at *k* = 50. As *k* decreased, the specificity declined only for LFK index down to 83%, 71%, and 65% at *k* = 20, 10, and 5, respectively. There was no decline for Egger test as it was anchored to its significance level (*p*‐value = 0.1) used for the threshold. In the meta‐analysis of “large” studies, similar results were observed (Table 2).

As *k* decreases, the contribution of random error to asymmetry increases, which is reflected in the specificity of the LFK index (Figure 2). This pattern is not observed in the Egger test because the *p‐*value is uniformly distributed in the no‐bias scenarios and there is no opportunity for increase or decrease in specificity since it is anchored to the predetermined significance level (i.e., 0.1), thus the specificity (1 – threshold) is always ∼0.90 regardless of changes in *k*.

### 4.3 Effect of *k*

**FIGURE 3:**
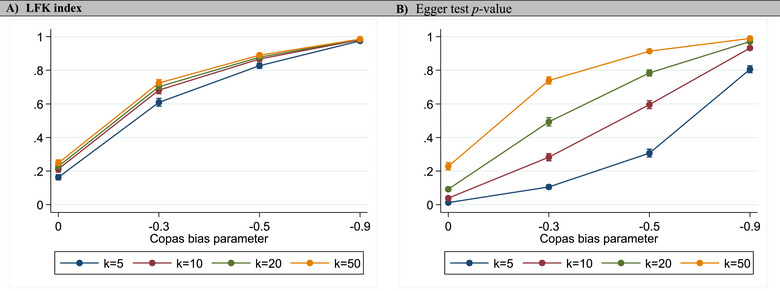
Margins plots for the probability of a positive resultin the smaller study sizes dataset based on cutoffs defined for (A) LFK index and (B) Egger test *p*‐values over different numbers of studies per meta‐analysis and different levels of the Copas bias parameter. This figure indicates that the variability in Table 2 for the LFK by *k* is just a function of random error but not so for Egger's *p*. The probability on the *y*‐axis indicates FP when *ρ* = 0 and TP when *ρ* ≠ 0. Similar trends were obtained for varying sample sizes of studies within meta‐analysis (results not shown).

The margins plots demonstrate that the proportion of meta‐analyses with LFK <‐1 did not vary substantially with *k*, but increased (as expected) with extent of bias (Figure 3). However, the proportion of meta‐analyses with Egger test *p‐*value <0.1 varies substantially with *k* as well as with extent of bias (Figure 3). This indicates that *p‐*value thresholds are not constant and are a function of *k*, which overrides its expected increase with the extent of bias. Therefore, the magnitude of *p‐*value informs us very little about the degree of (a)symmetry without factoring *k* into the interpretation, which is not the case with the LFK index.

### 4.4 Overall Interpretation

Following their simulation study, Schwarzer et al. drew several conclusions, which we contrast with our findings as reported above. To fully comprehend and interpret the simulation findings, it is crucial to understand that the LFK index and *p*‐value‐based methods’ performance is based on their ability to distinguish asymmetry due to addition of systematic error (in this simulation, due to publication bias) over and above existing asymmetry due to random error.

**Implication 1**: Schwarzer et al. stated, “The true positive rate [sensitivity] of the LFK index test (sic) was only larger compared with classic tests if the false positive rate [1‐specificity] was inflated.”

We demonstrate that this statement is false. The sensitivity (true‐positive rate) of the LFK index was consistently better than the Egger test, *regardless* of the false‐positive rate. The false‐positive rate for the LFK index decreased as *k* increased, and this is expected since random error, which contributes to false positives, diminishes as *k* increases (Figure 2). In contrast, the fixed specificity of the Egger test suggests that something other than random error is driving its false‐positive rate.

**Implication 2**: Schwarzer et al. state, “The false positive rate [specificity] of the LFK index test (sic) heavily depends on the number and sample size of studies in the meta‐analysis as well as the degree of between‐study heterogeneity.”

As outlined above, the increasing specificity of the LFK index with *k* is the expected behaviour of any quantitative method because random error‐related asymmetry decreases as *k* increases. The sample size statement was false since we could not replicate this in our simulation. The decrease in performance of the LFK index reported by Schwarzer et al. for “large” studies was probably due to simulation error on their part, stemming from minimal introduction of systematic error through the Copas selection model due to an erroneous and unrealistic distribution of total sample sizes (Figure 1). In addition, their suggestion that between‐study heterogeneity impacts the performance of the LFK index is also irrelevant in this context. Heterogeneity is a source of systematic error, and we ensured its absence in our simulated scenarios to isolate bias due to publication bias from other sources of bias.

**Implication 3**: Schwarzer et al. state, “The power [sensitivity] of classic tests was similar or larger than the LFK index test (sic) if the false positive rate [1‐specificity] of the LFK index test was used as significance level for the classic tests.”

What Schwarzer et al. refers here is that as *k* decreases, the *p‐value* cutoff should be increased to increase sensitivity (power). The sensitivity of the Egger test can increase by changing its cut‐off (e.g., from 0.1 to 0.2). However, doing so will also change its specificity, but will not change the discrimination of the test and therefore does not improve its overall performance (e.g., area under the curve). In addition, we have shown above that the change of interpretation of the *p*‐value with changes in *k* makes this strategy even more unrealistic and it is possible that such an interpretation by Schwarzer et al. could be considered an attempt at SPIN [22].

## 5 Recommendations for Evidence Synthesis

A summary of key recommendations is provided in Box 2, with detailed explanations presented below.

**Box 2. Recommendations for assessing the Doi plot (a)symmetry in meta‐analyses**

1. **Avoidance of *p*‐value‐based tests**
  - Avoid relying on *p*‐value‐based methods (e.g., Egger test) for assessing asymmetry.
  - These methods are heavily dependent on the number of studies (*k*).
2. **Visual interpretation**
  - Evaluate the symmetry, spread, and descent of the plot's limbs:
    - Unequal spread may suggest small‐study effects.
    - Unequal descent may indicate missing studies (publication bias).
    - Both features may co‐exist.
3. **Minimum number of studies (*k*)**
  - The Doi plot can be meaningfully interpreted with as few as five studies, unlike funnel plots, which typically require ten or more.
  - The LFK index remains interpretable at small *k* values, though visual interpretation of the Doi plot remains essential when *k* is low.
4. **Interpretation in context**
  - Interpret asymmetry within the context of study design and evidence base:
    - For randomized trials, asymmetry is more likely due to publication and/or reporting bias.
    - For observational studies, asymmetry is more likely due to small study effects (other biases, e.g., confounding, selection bias).
5. **Use of the Doi plot and LFK index**
  - Use the Doi plot for initial visual assessment of asymmetry.
  - Use the LFK index to quantify the degree of asymmetry:
    - LFK index < |1|: no asymmetry
    - LFK index between |1| and |2|: minor asymmetry
    - LFK index > |2|: major asymmetry

### 5.1 Interpreting Doi Plot Asymmetry

Researchers often struggle to accurately identify asymmetry using funnel plots [23], one proposed enhancement has been the inclusion of contour lines representing key statistical significance thresholds (e.g., *p*‐values = 0.01, 0.05, 0.1) to aid visual interpretation [24]. However, this approach is not without its limitations [25], and the threshold effect we demonstrate with *k* is still a challenge for such plots making Doi plots a better choice (Box 2 ***, Recommendation 1***). The latter is effectively addressed by the Doi plot, which eliminates the need for such enhancements.

The functionality of the Doi plot parallels the use of quantile–quantile (QQ) plots to check for data normality. Just as normality is often visually assessed by examining whether points in a QQ plot form a straight line, despite the availability of quantitative assessments with limited power [26].

The visual assessment of the Doi plot depends on the spread, descent, and number of studies on its two limbs. Three examples illustrate these concepts:

1. Small study effects: Both limbs have a similar number of studies and descend equally from the tip, but one limb is more spread out from the center line as study sizes decrease.
2. Publication bias: One limb has fewer studies and descends less from the tip, though its spread matches the other limb until its descent, indicating missing studies.
3. Combined effects: Both limbs descend equally, but one limb has fewer, more widely spread studies, indicating both small study effects and potential missing studies.

Thus, the visual assessment of Doi plot asymmetry remains critical when assessing asymmetry due to random or systematic error, as explained above (Box 2 ***, Recommendation 2***).

The Doi plot has several other advantages, it can be used even with five studies, as corroborated by the simulation results in this study. Nevertheless, systematic review authors should carefully distinguish between potential causes of Doi plot asymmetry and consider factors such as the intervention, its context, and variations in study design when interpreting findings. Furthermore, unlike funnel plots, the Doi plot imposes no restrictions on outcome scales, making it applicable to a wide range of metrics, including prevalence and standardized mean differences (Box 2 ***, Recommendations 3 & 4***) [12].

While the Doi plot and LFK index are also applicable to meta‐analyses of observational studies, effect estimates may become asymmetric due to decreases in quality (e.g., confounding and selection biases) of the primary studies. In such cases, asymmetry may reflect not just publication or reporting biases, but also differing degrees of other biases between smaller and larger studies (Box 2 ***, Recommendation 4***) [20]. To address this issue, especially in observational studies, some researchers advocate stratification by quality (higher vs. lower based on an arbitrary threshold) or risk of bias judgments, but this should be avoided as this can actually create a scenario that creates spurious within stratum asymmetry [27]. The use of quality or risk of bias assessments [28] to bias‐adjust meta‐analyses [29] may be a preferred approach.

### 5.2 LFK Index to Quantify Doi Plot Asymmetry

The LFK index is a quantitative measure of Doi plot (a)symmetry and quantifies both the numbers and distance of studies on either side of the largest study. It aims to provide an objective and reproducible quantitative measure to assess (a)symmetry and, more importantly, provides a quantitative threshold when formal decision‐making requires a binary determination of asymmetry. It is also easily able to be used in comparative analyses and therefore, together with the Doi plot, offers a comprehensive approach to assessing publication bias in evidence syntheses. As the LFK index progressively deviates from 0, it denotes asymmetry due to (a) random error (asymmetry by chance), (b) systematic error (publication bias being one of the reasons), or (c) a combination of systematic and random error. Although the threshold ±1, has been set for that beyond which the observed asymmetry is more likely due to systematic error rather than random error, the overlap only diminishes markedly beyond LFK ±3 (Box 2 ***, Recommendation 5***).

The LFK index versus the *p*‐value‐based methods for publication bias have distinct performance profiles. First, the LFK index is independent of *k* while the *p*‐value‐based methods are not and is therefore a moving goalpost for (a)symmetry. With few studies, the random error overwhelms *p‐*value‐based methods at the traditional cut‐off, but modifying this cut‐off would increase false‐positive rates without improving its discriminative performance.

The limitation of the *p‐*value‐based method for quantifying asymmetry have thus far remained unrecognized resulting in numerous statistical updates to *p*‐value‐based methods [20, 30]. Given the threshold dependence on *k*, these alternatives have not had a better performance than the Egger test, as acknowledged in a highly cited paper [31]. Sterne et al. also recommend against using *p‐*value‐based methods for funnel plot asymmetry when there are fewer than 10 studies in a meta‐analysis [31] as that is when the conventional 0.1 cut‐off markedly fails. This limitation is not a concern for the LFK index, as its threshold is independent of *k*. Sterne et al. further caution against using asymmetry tests when the standard errors of intervention effect estimates are similar (i.e., when studies are of similar sizes). This limitation was highlighted by the flawed simulation of “large” trials by Schwarzer et al., where most studies were of comparable size, resulting in exceedingly low sensitivity [31]. While it has previously been recommended that tests for funnel plot asymmetry should be used in only a minority of meta‐analyses [32], the same recommendation does not apply to the LFK index.

## 6 Conclusion

Our study highlights the robustness and practicality of the Doi plot and LFK index in assessing publication bias in meta‐analyses. Unlike *p*‐value‐based methods, the LFK index performs consistently across varying numbers of studies, effectively distinguishing between asymmetry caused by random error and systematic error (e.g., due to publication bias). Criticisms raised by Schwarzer et al., particularly with respect to large studies, appear to stem from simulation design choices and interpretation rather than a reflection of the LFK index performance. The absence of limitations regarding the choice of effect measures and the number of studies in a meta‐analysis underscores the need for a widespread transition to the Doi plot and LFK index‐based assessment system for publication bias, offering a more flexible and reliable alternative to the current standard. While there is certainly scope for further exploration of these novel methods in complex meta‐analyses with high heterogeneity, the basic foundations for a change in approach have now been laid down.

## Conflicts of Interest

LFK and SARD developed the Doi plot and LFK index. XR and PK have no conflicts of interest to declare.

## Supporting information




**Supplementary File 1**: jebm70063‐sup‐0001‐SuppMat.zip
